# False reassurances based on inadequate data not helpful

**DOI:** 10.1017/S0033291725102092

**Published:** 2025-10-10

**Authors:** Mark A. Horowitz, Michael P. Hengartner, Martin Plöderl, Joanna Moncrieff

**Affiliations:** 1 Research and Development Department, North East London NHS Foundation Trust (NELFT), Essex, UK; 2 Department of Applied Psychology, Kalaidos University of Applied Sciences, Zurich, Switzerland; 3 University Clinic of Psychiatry, Psychotherapy, and Psychosomatics, Paracelsus Medical University, Salzburg, Austria; 4 Division of Psychiatry, University College London, London, UK

We welcome Baethge, Bschor, and Henssler’s (2025) acknowledgment that antidepressant withdrawal is a serious clinical problem. However, to claim that withdrawal symptoms are not frequent and rarely severe, based on inadequate, short-term and methodologically weak studies is misleading to clinicians and patients.

We are accused of having double standards, but even weak evidence for harms must be taken seriously as it is more important than efficacy. When uncertainty exists regarding safety, we should err on the side of caution. For decades, guidelines have claimed that withdrawal symptoms are mild and brief, based on short trials never designed to assess withdrawal (Horowitz et al., 2025). This led to a lack of informed consent at initiation and disbelief when patients later experienced distressing symptoms on stopping. Henssler et al.’s (2024) meta-analysis perpetuates these problems by re-examining the same short, inadequate studies.

Baethge et al. defend this work, yet several of their central claims are not supported by evidence and their conclusions that withdrawal affects only one in six patients, with 3% experiencing severe symptoms are not reliable.

## Placebo as a comparator

Baethge et al. argue that placebo withdrawal should be subtracted from antidepressant withdrawal to obtain a ‘true’ incidence. While we agree that comparison with a control group is generally useful, this approach is flawed here because it ignores severity. Everyday symptoms such as headache or insomnia occur in people stopping placebo but are unlikely to be as severe or persistent as the potentially disabling syndromes seen after antidepressant withdrawal.

Henssler’s data confirm this. Although their methods underestimate its frequency, severe withdrawal was five times more likely to occur after stopping antidepressants than placebo (Henssler et al., 2024). Furthermore, numerous patient reports testify to the potentially severe nature of withdrawal symptoms, which can sometimes be disabling, leading to job loss, relationship breakdown, and physical accidents (Guy, Brown, Lewis, & Horowitz, 2020; Moncrieff, Read, & Horowitz, 2024).

Unfortunately, withdrawal measures, such as the Discontinuation Emergent Signs and Symptoms (DESS), only assess the presence of symptoms and not their severity. This contrasts with mood rating scales used to assess efficacy, such as the Hamilton Rating Scale for Depression (HRSD), which rates symptoms on a Likert scale of absent/minimal to very severe. Therefore, subtraction of placebo withdrawal likely obscures clinically important differences and undermines the reliability of the ‘one in six’ estimate.

## Duration of use and withdrawal risk

Baethge et al. suggest the link between treatment duration and withdrawal risk is ‘inconclusive’. This claim is implausible. Neurobiology provides a clear rationale for withdrawal. Chronic Selective Serotonin Reuptake Inhibitors (SSRI) and Serotonin-Noradrenaline Reuptake Inhibitor (SNRI) use leads to receptor downregulation and widespread neuroadaptations. The longer the exposure, the more entrenched these changes become, and the greater the disruption when the drug is withdrawn (Horowitz, Framer, Hengartner, Sørensen, & Taylor, 2023). Antidepressants are not exempt from the basic pharmacological principles that govern all psychoactive drugs.

Although there was no association between duration of prior use and incidence of withdrawal in the meta-regression analysis by Henssler et al., most included trials involved prior use of less than 6 months. This restricted variability in treatment duration biases its correlation with other variables toward zero (floor effect) – akin to concluding that age does not correlate with developing dementia, based on people who are 20–30 years old. This applies to another recent meta-analysis (Kalfas et al., 2025) where the longest trial included in the analysis involved agomelatine, an antidepressant with no known risk of withdrawal, and several short trials included paroxetine and venlafaxine, which are of high risk, thus creating the appearance of an inverse correlation. The Henssler meta-regression also included studies with numerous different medications, tapering periods and assessment procedures, which is likely to obscure any effect (the ecological fallacy). The Zhang meta-analysis found a trend relationship between the duration of use and risk of withdrawal, although this analysis was also likely limited by short-term trials and a similar floor effect.

Survey data also suggest that the duration of prior use is highly relevant. One large study of users of the UK’s public therapy service found patients treated for more than 2 years had 10-fold higher odds of withdrawal and five-fold higher odds of severe withdrawal than those treated for less than 6 months (Horowitz et al., 2025). While survey self-selection may inflate absolute incidence, such a dramatic gradient is unlikely to be artefactual.

In the UK, over 4 million people have taken antidepressants for >2 years; in the United States, 25 million. It is not valid to generalize findings from trials that mainly last from 8 to 26 weeks to this population.

## Systematic versus spontaneous assessment

Baethge et al argue that by choosing a narrow range of studies, we excluded most of the evidence base. This is correct, but deliberate. The majority of trials in Henssler’s review only reported spontaneous adverse events in efficacy studies, where withdrawal detection was not an objective. It is well documented that adverse events are usually neither systematically assessed nor reliably reported (Phillips, Hazell, Sauzet, & Cornelius, 2019), and therefore, such methods are unfit for purpose.

For this reason, we conducted a re-analysis, based on the small number of studies that employed a systematic measure of withdrawal symptoms. We acknowledged that none of these studies included a placebo comparison group, which may have inflated estimated withdrawal effects. On the other hand, four of these five studies lasted for 3 months or less and one involved agomelatine, and hence, they may have underestimated the risks associated with medium- or long-term use of other antidepressants.

Henssler et al. (2024) did not include several higher-quality, informative studies involving people who had used antidepressants for longer periods. These suggest higher rates of withdrawal symptoms. Rosenbaum, Fava, Hoog, Ascroft, and Krebs (1998) examined 300 patients treated for an average of 11 months, using the DESS with comparison to a continuing-treatment control group, and found withdrawal in a week of observation in 66% of those stopping paroxetine, 60% in those stopping sertraline, and only 14% with the long-acting fluoxetine acting as a control of sorts (though a proportion of this will be genuine withdrawal, since withdrawal occurs within a week of cessation for some), suggesting about half of longer term users experience withdrawal (Rosenbaum et al., 1998). Another brief treatment-interruption trial also documented similar rates of withdrawal along with significant impairment after a year of use (Michelson et al., 2000).

## Other reviews

Baethge et al. make a number of criticisms of the review by Zhang et al (2024) and also cite the recent review by Kalfas et al. to support their conclusions. However, whereas Zhang et al. included data from some studies involving longer-term users, the main analysis in the Kalfas et al.’s review was based on 11 studies, 10 of which involved people who had taken antidepressants for 3 months or less, and the other study involved agomelatine which is recognized to have a low risk for withdrawal effects. Despite this, and despite the fact that withdrawal symptoms were not rated for severity, the analysis provided clear evidence that withdrawal symptoms are more common following the cessation of antidepressants compared to placebo (Kalfas et al., 2025). Additionally, the Kalfas et al.’s review under-estimated the incidence of individual withdrawal symptoms by relying on spontaneous reporting of adverse effects (Antidepressant withdrawal symptoms: Improvements needed in clinical research, 2025).

Baethge et al. criticize us for preferring a smaller number of well-conducted studies to a larger number of imperfect studies. But quantity does not make up for quality and extrapolating findings from poorly conducted, short-term studies not designed to examine withdrawal to direct the management of tens of millions of people using antidepressants is not responsible or valid.

## References

[r1] Hengartner MP, Plöderl M, Moncrieff J (2025). Antidepressant withdrawal symptoms. Improvements needed in clinical research. BMJ, in press.

[r2] Baethge, C., Bschor, T., & Henssler, J. (2025). Double counting, double standards. Psychological Medicine, in press. 10.1017/S0033291725101980 PMC13054895 41165089

[r3] Guy, A., Brown, M., Lewis, S., & Horowitz, M. (2020). The ‘patient voice’: Patients who experience antidepressant withdrawal symptoms are often dismissed, or misdiagnosed with relapse, or a new medical condition. Therapeutic Advances in Psychopharmacology, 10, 2045125320967183. 10.1177/2045125320967183. 33224468 PMC7659022

[r4] Henssler, J., Schmidt, Y., Schmidt, U., Schwarzer, G., Bschor, T., & Baethge, C. (2024). Incidence of antidepressant discontinuation symptoms: A systematic review and meta-analysis. The Lancet. Psychiatry, 11(7), 526–535. http://doi.og/10.1016/S2215-0366(24)00133-0. 38851198 10.1016/S2215-0366(24)00133-0

[r5] Horowitz, M., Buckman, J., Saunders, R., Aguirre, E., Davies, J., & Moncrieff, J. (2025). Antidepressants withdrawal effects and duration of use: A survey of patients enrolled in primary care psychotherapy services. Psychiatry Research, 116497. 10.1016/j.psychres.2025.116497. 40404538

[r6] Horowitz, M., Framer, A., Hengartner, M. P., Sørensen, A., & Taylor, D. (2023). Estimating risk of antidepressant withdrawal from a review of published data. CNS Drugs, 37(2), 143–157. 10.1007/s40263-022-00960-y. 36513909 PMC9911477

[r7] Kalfas, M., Tsapekos, D., Butler, M., McCutcheon, R. A., Pillinger, T., Strawbridge, R., … Jauhar, S. (2025). Incidence and nature of antidepressant discontinuation symptoms: A systematic review and meta-analysis. JAMA Psychiatry (Chicago, IL). 10.1001/jamapsychiatry.2025.1362. PMC12242823 40632531

[r8] Michelson, D., Fava, M., Amsterdam, J., Apter, J., Londborg, P., Tamura, R., & Tepner, R. G. (2000). Interruption of selective serotonin reuptake inhibitor treatment. Double-blind, placebo-controlled trial. The British Journal of Psychiatry: The Journal of Mental Science, 176, 363–368. 10.1192/bjp.176.4.363. 10827885

[r9] Moncrieff, J., Read, J., & Horowitz, M. A. (2024). The nature and impact of antidepressant withdrawal symptoms and proposal of the Discriminatory Antidepressant Withdrawal Symptoms Scale (DAWSS). Journal of Affective Disorders Reports, 16, 100765. 10.1016/j.jadr.2024.100765.

[r10] Phillips, R., Hazell, L., Sauzet, O., & Cornelius, V. (2019). Analysis and reporting of adverse events in randomised controlled trials: a review. BMJ Open, 9(2), e024537. 10.1136/bmjopen-2018-024537. PMC6398660 30826796

[r11] Rosenbaum, J. F., Fava, M., Hoog, S. L., Ascroft, R. C., & Krebs, W. B. (1998). Selective serotonin reuptake inhibitor discontinuation syndrome: A randomized clinical trial. Biological Psychiatry, 44(2), 77–87. 10.1016/S0006-3223(98)00126-7. 9646889

[r12] Zhang, M.-M., Tan, X., Zheng, Y.-B., Zeng, N., Li, Z., Horowitz, M. A., … Li, S.-X. (2024). *Incidence and risk factors of antidepressant withdrawal symptoms: A meta-analysis and systematic review.* 1–12. https://scholar.google.com/citations?view_op=view_citation&hl=en&citation_for_view=sEcbBuUAAAAJ:uWQEDVKXjbEC 10.1038/s41380-024-02782-4 39394455

